# Common themes in evoked ion channel translocation in neuroplastic and homeostatic plasticity

**DOI:** 10.3389/fphar.2026.1727525

**Published:** 2026-03-30

**Authors:** Kirk D. Haan, Emma Gaudet, Thomas E. Fisher

**Affiliations:** Department of Anatomy, Physiology, and Pharmacology, College of Medicine, University of Saskatchewan, Saskatoon, SK, Canada

**Keywords:** AMPA, endophilin, ion channel, long-term potentiation, osmoregulation, SNARE, translocation, TRPV1

## Abstract

Ion channel trafficking to and from the plasma membrane is an essential adaptation of neurons to external stimulation, and these distinct processes are tightly regulated by various intrinsic and extrinsic factors. The translocation of ion channels to the plasma membrane can strengthen synaptic connections to enhance and sustain learning and memory. Ion channel translocation can also enhance homeostatic regulation through the creation and maintenance of negative feedback loops. The removal of ion channels from the plasma membrane is critical for preventing hyperexcitability and for achieving homeostatic balance (either of activity or inactivity). Recently described and characterized mechanisms of ion channel exocytosis and endocytosis suggest that a select number of proteins, including specific SNARE isoforms and endophilin, are essential for regulating multiple types of evoked ion channel translocation. Moreover, dysfunction of these key proteins is implicated in the pathophysiology of neurodegenerative diseases and may underly common mechanisms of disease development and progression. This review highlights recent discoveries related to the regulation of evoked ion channel translocation and provides novel insights on how the shared components may contribute to normal functioning and disease states.

## Introduction

Neurons must adjust their level of excitability to cope with different physiological situations, and can accomplish this by modulating the numbers and types of ion channels present on the plasma membrane (PM) (Misonou et al., 2004; Lai and Jan, 2006). The movement of ion channels to and from the PM can either be constitutive (i.e., continuous and unregulated) or evoked (i.e., some stimulus activates the process). The evoked, reversible trafficking of ion channels is an important component of the long-term modulation of neuronal activity (Cerny and Huber, 2011; Rapoport et al., 2017). One of the earliest characterized examples of evoked ion channel translocation is in the neuroendocrine bag cell neurons of *Aplysia californica*, which undergo a sustained increase in excitability known as an afterdischarge (Conn and Kaczmarek, 1989). Once an afterdischarge is triggered, the bag cell neurons fire continuously and rapidly for 20–30 min and release a hormone known as egg-laying hormone, which initiates a series of stereotyped behaviours in the animal culminating in the laying of eggs (Conn and Kaczmarek, 1989). This state of enhanced excitability is caused in part by an increase in voltage-gated Ca^2+^ currents (Strong et al., 1987), which is mediated by the translocation of voltage-gated Ca^2+^ channels (VGCCs) from internal storage sites to the PM (Zhang et al., 2008). Since that time, several other examples of the regulation of neuronal excitability by ion channel translocation have been identified. These examples occur in various neuron types and in a variety of neuronal compartments. Evoked ion channel translocation is an example of non-secretory exocytosis (Hille and Catterall, 2012), and is usually mediated by sets of proteins that are distinct from those that mediate secretory release (Hille and Catterall, 2012; Worley et al., 2007). Understanding the mechanisms underlying evoked ion channel translocation is essential for a full understanding of how these processes regulate neuronal function. This review illustrates some examples of ion channel translocation in neuroplastic and homeostatic plasticity. It also discusses some of the commonalities and differences between the mechanisms in those examples.

Ion channels are translocated to the PM in response to a variety of stimuli. Neuroplastic translocation involves the strengthening of synapses to sustain an electrical communication pathway and is commonly used for processes like learning and memory (Lynch, 2004; Pastalkova et al., 2006). In some neuron types, such as hippocampal and basal forebrain neurons, α -amino-3-hydroxy-5-methyl-4-isoxazole propionic acid-type glutamate receptors (AMPARs) are rapidly translocated to the PM from the postsynaptic density (PSD) in response to repetitive electrical stimulation, a phenomenon known as long-term potentiation (LTP) (Sumi and Harada, 2020). Homeostatic plasticity involves the temporary modulation of neuronal activity (e.g., excitability) to promote a return to a previous homeostatic equilibrium (Cerny and Huber, 2011; Williams et al., 2001; Rosati and McKinnon, 2004). This modulation of neuronal activity can be mediated by post-translational modifications that alter ion channel open probability or by the translocation of ion channels to the PM to enhance firing (Dubyak, 2004; O’Leary, 2018). The excitability of central and peripheral neurons is often determined by voltage-gated Na^+^ channels (VGSCs) in the somata and axons (Solé and Tamkun, 2020; Chahine et al., 2005; Davis, 2013). VGSC-mediated regulation of excitability can be accomplished through both the regulation of VGSC opening at the PM and by VGSC translocation to and from the PM (Solé and Tamkun, 2020; Chahine et al., 2005; Davis, 2013).

A well-studied example illustrating the importance of ion channel translocation in homeostatic plasticity involves the regulation of excitability in the magnocellular neurosecretory cells (MNCs) of the hypothalamus in response to osmotic stimuli. MNCs regulate body fluid and electrolyte balance (i.e., osmolality) by transducing increases in extracellular fluid osmolality into an increase in the firing of action potentials (APs) (Sharif Naeini et al., 2006; Zaelzer et al., 2015). Increased AP firing in MNCs enhances vasopressin (VP) release (Stern and Armstrong, 1998; Oliet and Bourque, 1992), which then enhances systemic vasal tone and renal water reabsorption (Robertson, 1983). Further increases in osmolality are buffered by the water reabsorption to maintain homeostatic equilibrium (Robertson, 1983). The sustained release of VP by MNCs (e.g., in response to water deprivation or salt loading) is accomplished by various structural and functional changes, including the hypertrophy of MNC somata (Shah et al., 2014; Haan and Fisher, 2023; Prager-Khoutorsky, 2021), and the translocation of ion channels (Haan et al., 2023; Sharma et al., 2017). Under hyperosmotic conditions, osmotically-induced ion channel translocation enhances MNC excitability to osmotic stimuli, which helps to sustain increased AP firing and VP release while the osmotic stimulus is still present (Haan et al., 2023; Sharma et al., 2017). Increased osmolality also triggers other osmosensitive central neurons to activate the sensation of thirst (Gizowski and Bourque, 2018), which leads to the ingestion of fluid. This causes a reduction in serum osmolality, a decrease in MNC firing, and a decrease in VP output (Robertson, 1983; Zerbe and Robertson, 1983). The decrease in MNC firing also leads to a reversal of both hypertrophy and ion channel translocation to the MNC plasma membrane (Shah et al., 2014; Haan et al., 2023).

The translocation of ion channels from the PM to internal storage sites—otherwise known as channel internalization—can also be constitutive or triggered (Estadella et al., 2020; Hu et al., 2024). Multiple mechanisms for ion channel internalization exist, and the mechanism used depends on several factors, including whether ion channel internalization is constitutive or triggered, the location of the intracellular destination(s) of the internalized ion channels, and, the type of stimulus (e.g., neuroplastic or homeostatic neuroplasticity) involved (Estadella et al., 2020; Hu et al., 2024). Soluble N-ethylmaleimide-sensitive factor attachment protein receptors (SNAREs) play essential roles in both exocytosis and endocytosis (Jahn et al., 2024), including in the exocytosis and endocytosis of ion channels (Planells-Cases et al., 2011). Many other proteins may be involved in ion channel internalization, including clathrin, dynamin, and caveolin (Hansen and Nichols, 2009; Chaudhary et al., 2014). The endocytic protein endophilin was recently shown to be integral to various endocytic processes, and it may govern multiple types of ion channel endocytosis (Kjaerulff et al., 2011; Casamento and Boucrot, 2020). In neuronal long-term depression (LTD) (Bear and Abraham, 1996; Collingridge et al., 2010), ion channels and other membrane proteins are internalized following a reduction in excitatory stimuli (Bear and Abraham, 1996; Collingridge et al., 2010). As noted above, ion channel internalization in MNCs is part of the recovery from the hypertrophied state (Haan et al., 2023). The internalization of dynorphin cell surface receptors also plays an important role in the regulation of MNC firing. For example, somatodendritic VP and dynorphin release act in an autocrine fashion to negatively regulate MNC firing (Brown and Bourque, 2004). The triggered internalization of dynorphin receptors from the MNC PM restores normal firing patterns and maintains the axonal VP release required for osmoregulation (Stern and Armstrong, 1998; Ludwig, 1998; Ludwig et al., 2005). However, the endocytic machinery governing triggered internalization in MNCs remains unknown. These examples illustrate the importance of ion channel internalization for the regulation of neuronal excitability.

Although the purposes of neuroplastic and homeostatic plasticity are fundamentally different, multiple similarities between the mechanisms of ion channel transport to and from the PM have been identified. Newly characterized pathways for the regulation of ion channel translocation by proteins like endophilin and synaptotagmin-11 (Syt11) have been investigated, and this research has provided new insights into the shared pathways. This review highlights similarities in the mechanisms of exocytosis and endocytosis in the various forms of evoked ion channel translocation in neuroplastic and homeostatic plasticity, especially those involving AMPARs/VGCCs and TRPV1 channels, respectively. This review also highlights how these similarities, especially in the shared usage of endophilins and Syt11, may be implicated in neuronal dysfunction and disease.

## Key intracellular components of evoked ion channel translocation

SNAREs are a superfamily of proteins responsible for most exocytic and endocytic processes in neurons (Jahn et al., 2024). SNARE proteins enable intracellular vesicles to fuse with the PM, and they ensure that this process occurs in a highly specific and highly regulated manner (Jahn et al., 2024). SNARE complex assembly involves the interactions of 3–4 specific proteins (e.g., synaptobrevin, SNAP-25, synaptotagmin, syntaxin) located on the cytosolic side of the PM or on intracellular vesicles, and it is essential for both exocytic and endocytic movement (Vivona et al., 2013; Xu et al., 2013). SNAP-25 and syntaxin are typically located on the PM, while synaptotagmin and synaptobrevin are located on the vesicle to undergo exocytic fusion (Jahn et al., 2024). Each SNARE protein possesses multiple isoforms that are optimized for the trafficking method, type of cargo being transported, and trafficking speed (Jahn et al., 2024). While Ca^2+^-dependent SNAREs are typically involved in faster processes like neurotransmitter release (Wolfes and Dean, 2020), Ca^2+^-independent SNAREs typically mediate slower processes like non-secretory exocytosis and the release of certain somatodendritic substances (Jahn et al., 2024; Wolfes and Dean, 2020).

**FIGURE 1:**
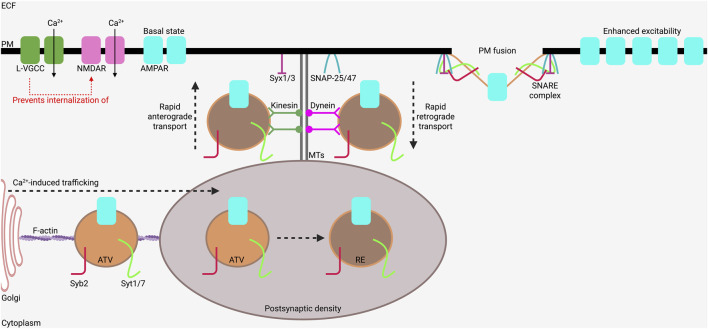
SNARE-mediated AMPAR trafficking to the plasma membrane. Ca^2+^ influx through L-VGCCs and NMDARs enhance the trafficking of AMPARs to boost synaptic strength during processes like learning and memory. The presence of L-VGCCs prevents internalization of NMDARs, which enhances Ca^2+^ influx in neurons. AMPARs undergo maturation in the Golgi and travel along filamentous actin in AMPAR trafficking vesicles (ATVs) which also contain synaptotagmin-1 and 7 (Syt1/7) and synaptobrevin-2 (Syb2). Once they reach the actin-rich post-synaptic density (PSD), they are trafficked in recycling endosomes (REs), which then rapidly transport AMPARs, Syt1/7, and Syb2 to the plasma membrane (PM) along microtubules (MTs). Syntaxin-1 or -3 (Syx1/3) and SNAP-25 or -47 are PM-bound SNARE proteins that mediate the fusion of AMPAR-containing REs with the PM through their interactions with Syt1/7 and Syb2. AMPAR-containing REs cycle between the PM and the PSD to change excitability. Figure created using Biorender.com.

Perhaps the most well-described example of channel translocation as a long-term adaptation to stimuli is LTP. LTP occurs when repeated, high-intensity, depolarizing stimuli strengthen a particular synaptic connection (Malenka and Bear, 2004). This process is dependent on the translocation of AMPARs to the post-synaptic PM to enhance neuronal excitability and the likelihood for signal propagation (Derkach et al., 2007). These ligand-gated ion channels are activated by glutamate binding to mediate fast excitatory neurotransmission (Traynelis et al., 2010). Hence, AMPAR translocation to the PM at post-synaptic locations enhances fast synaptic transmission (Mihalas et al., 2021; Collingridge et al., 2004; Bassani et al., 2013). Importantly, exocytosis of AMPARs was demonstrated to be dependent on SNARE mechanisms (Lledo et al., 1998; Arendt et al., 2015; Jurado et al., 2013). When AMPARs leave the Golgi, they travel primarily in AMPAR-trafficking vesicles (ATVs) (Lledo et al., 1998; Peters et al., 2021). The membranes of ATVs contain synaptotagmin-1 (Syt1) and synaptotagmin-7 (Syt7) (Wu et al., 2017), as well as synaptobrevin-2 (Peters et al., 2021). Syt1 and Syt7 are highly Ca^2+^-dependent (Wolfes and Dean, 2020), and the rapid influx of Ca^2+^ caused by depolarization rapidly activates these proteins to initiate translocation of ATVs to the PM (Peters et al., 2021; Henley et al., 2011). The ATVs are then trafficked along actin filaments to the PSD (Cao et al., 2023), which is an actin-rich area that assists in the sorting of post-synaptic receptors (Kim and Sheng, 2009; Boeckers, 2006). Here, they interact with the membrane SNARE proteins syntaxin-1 or syntaxin-3 and SNAP-25 or SNAP-47 (Jurado et al., 2013), leading to exocytic fusion with the PM and AMPAR insertion. AMPARs can be rapidly cycled between the PM and the PSD via recycling endosomes to rapidly change excitability (Esteves da Silva et al., 2015). This process requires intact microtubules (MTs) (Esteves da Silva et al., 2015; Zhang et al., 2021). The different steps of the AMPAR trafficking process are illustrated in Figure 1.

VGCCs are also involved in LTP maintenance (Atlas, 2013; Berger and Bartsch, 2014). L-type VGCCs (L-VGCCs) were recently shown to indirectly regulate activity-dependent exocytosis of AMPARs in hippocampal neurons (Hiester et al., 2017). The study by Hiester et al. showed that L-VGCCs prevented PM-bound N-methyl-D-aspartate receptors (NMDARs)—an ionotropic nonselective cation channel involved in LTP (Lüscher and Malenka, 2012)—from being internalized, which then led to an increase in AMPAR translocation to the PM and LTP (Hiester et al., 2017). This process is also depicted in Figure 1. Moreover, L-VGCC stimulation with Bay K8644, a specific L-VGCC agonist, in the absence of NMDA caused AMPAR internalization (Hiester et al., 2017). The results of this study are consistent with multimodal regulation of AMPAR insertion by L-VGCCs. N-type and P/Q-type VGCCs (N-VGCCs and P/Q-VGCCs, respectively) have also been shown to play key roles in the presynaptic release of glutamate onto post-synaptic neurons containing AMPARs (Saghian and Wang, 2022). Depolarization and Ca^2+^ influx at the presynaptic terminal causes Syt1-mediated exocytic fusion of synaptic vesicles (SVs) containing N-VGCCs (along with SNAP-25 and syntaxin-1) on the PM (Saghian and Wang, 2022; Frank, 2014; He et al., 2018; Lévêque et al., 1994; Sheng et al., 1994). This process enhances depolarization and facilitates glutamate release onto the post-synaptic neuron (Saghian and Wang, 2022; Frank, 2014; He et al., 2018). Although N-VGCC and AMPAR translocation to the PM are in different parts of the synapse (i.e., N-VGCCs in the presynaptic cell, AMPARs in the post-synaptic cell), these processes utilize the same SNARE machinery during fusion with the PM (Jurado et al., 2013; Saghian and Wang, 2022; Frank, 2014; He et al., 2018).

As outlined in the introduction, ion channel translocation helps regulate excitability in MNCs in response to osmotic stimuli. MNCs respond to minute-to-minute changes in osmolality via activation of ΔN-TRPV1, a mechanosensitive N-terminal variant of the transient receptor potential vanilloid type-1 channel (Zaelzer et al., 2015; Ciura and Bourque, 2006). ΔN-TRPV1 transduces hyperosmotic stimuli into cell shrinkage-induced cation influx and depolarization (Oliet and Bourque, 1993; Bourque et al., 2002), which increases AP firing and VP release from the axon terminal located in the posterior pituitary (Bourque, 2008). MNCs possess a unique and densely interwoven cytoskeletal scaffold of submembranous actin and MTs (Prager-Khoutorsky et al., 2014). This dense cytoskeletal structure is essential for the mechanosensitivity of MNCs during osmotically induced cell shrinkage (Prager-Khoutorsky et al., 2014; Zhang and Bourque, 2008; Zhang Z. et al., 2007; Prager-Khoutorsky and Bourque, 2015; Prager-Khoutorsky and Bourque, 2010). ΔN-TPRV1 has been shown to interact directly with MTs, contributing to their mechanosensitivity (Prager-Khoutorsky et al., 2014). Actin density and MT density increase during chronic hyperosmotic stimulation, and this is thought to enhance the MNC response to osmotic stimuli (Park et al., 2021; Prager-Khoutorsky and Bourque, 2010; Zhang and Bourque, 2008). Recent work has identified small (0.6 μm in diameter) fenestrations in the submembranous actin layer, and it is there that ΔN-TRPV1 and MTs preferentially interact (Murtaz and Bourque, 2025). Acute increases in osmolality also cause membranous pits to form at these fenestrations (Murtaz and Bourque, 2025). Based on these results, it can be postulated that ΔN-TRPV1 activation is triggered at these sites. Moreover, the fenestrations may be the sites at which ion translocation occurs. Translocation of ΔN-TRPV1 channels to the PM has been demonstrated in isolated rat MNCs after treatment with hyperosmotic saline or after 24 h of water deprivation (Haan et al., 2023). The resulting translocation was shown to require SNARE-dependent exocytic fusion with the PM, as treatment with inhibitors of the SNARE complex (i.e., Exo-1 and TAT-NSF700) prevented ΔN-TRPV1 translocation (Haan et al., 2023). While their exact function remains unknown, the actin fenestrations in MNCs may play a role in the regulation of ΔN-TRPV1 activity or in the osmotically induced translocation of ion channels.

Channels other than ΔN-TRPV1 have also been hypothesized to undergo translocation to the PM of MNCs in response to chronic hyperosmotic stimuli. The addition of salt to the drinking water of rats (i.e., salt loading) for 7 days increased the synthesis and expression of Na_V_1.2 and Na_V_1.6 (Tanaka et al., 1999); changes in the expression of these two VGSC subtypes were identified through electrophysiology and *in situ* hybridization (Tanaka et al., 1999). This increase in the functional expression of VGSCs in MNCs in response to salt loading likely alters their excitability and osmosensitivity. The authors postulated that the significant increase observed in VGSC subunit expression, combined with the significant increase in Na^+^ currents observed after salt loading, demonstrates that VGSCs underwent osmotically-induced translocation (Tanaka et al., 1999). However, this was not confirmed by immunostaining at the cellular level; the authors only reported immunoreactivity at the level of the SON, not of individual MNCs. Salt loading also increased the functional expression of ENaCs on the PM, and possibly their translocation to the PM from intracellular stores (Sharma et al., 2017). ENaC translocation in MNCs has been postulated to help maintain a more depolarized resting membrane potential and thereby increase osmosensitivity (Tasker et al., 2020). The mechanisms of ENaC translocation have already been extensively studied in other cell types. In oocytes, for example, ENaC fusion with the PM was shown to be regulated by syntaxin-1A, synaptobrevin-2, and SNAP-23 (Saxena et al., 2006; Butterworth et al., 2005; Condliffe et al., 2003; Kusche-Vihrog et al., 2009). ENaC translocation has also been extensively studied in other non-neuronal cell populations (Morachevskaya and Sudarikova, 2021; Butterworth, 2010; Alli et al., 2015). The translocation of ΔN-TRPV1 in MNCs, and possibly that of other channels, including VGSCs (Tanaka et al., 1999), ENaC (Sharma et al., 2017), and VGCCs (Zhang W. et al., 2007), may be part of a larger set of structural and functional adaptations to chronic osmotic stress required to maintain homeostatic VP release. Interestingly, the SNARE machinery governing exocytic and endocytic processes has been shown to differ between the somata and the axons of MNCs (Miyata et al., 2001; Tobin et al., 2012). Although the exact identities of the machinery are currently unknown, a study of the specific machinery used in each subcellular compartment could further our understanding of the specific pathways involved in MNC regulation.

## Commonalities in ion channel internalization

**FIGURE 2:**
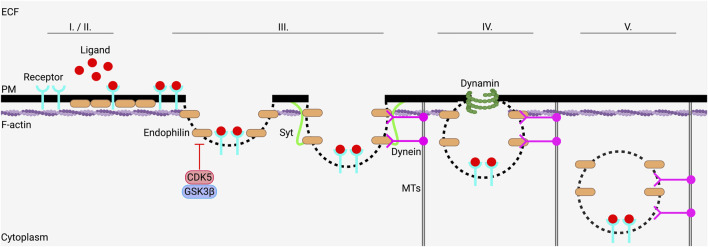
FEME requires the complex coordination of multiple protein interactions to internalize cargo. FEME is initiated through an internalizing stimulus such as specific ligand-receptor binding interactions (I), which is followed by the enrichment and activation of endophilin (II). Next, endophilin-positive assemblies (EPAs) are created, with complex interactions between actin, endophilin, synaptotagmin (Syt), and dynein being required for appropriate EPA formation. The scission of EPAs is accomplished by dynamin (IV), after which dynein carries EPAs on microtubules (MTs) from the plasma membrane (PM) to early endosomes (V). (II) and (III) are inhibited by CDK5 and GSK3 β . Figure created using Biorender.com.

SNARE proteins also play a role in ion channel endocytosis, especially Syt11 (Estadella et al., 2020; Jahn et al., 2024). The endocytic protein endophilin has recently been shown to be a key mediator of various endocytic processes (Boucrot et al., 2015; Bai et al., 2010; Renard et al., 2015; Chen et al., 2003). Fast endophilin-mediated endocytosis (FEME) is a clathrin-independent endocytic process whose primary regulator is the protein endophilin (Casamento and Boucrot, 2020; Boucrot et al., 2015). Endophilin possesses multiple isoforms, but the most well-studied in FEME are endophilin-A1 (EndoA1) and endophilin-A2 (EndoA2) (Kjaerulff et al., 2011). FEME is not a constitutively active process, but instead is rapidly activated through receptor-ligand interactions or the dephosphorylation of proteins within endophilin-positive assemblies (EPAs) (Casamento and Boucrot, 2020; Boucrot et al., 2015; Ferreira et al., 2021). FEME utilizes retrograde motor proteins called dyneins to transport EPAs carrying cargo to intracellular targets along MTs (Ferreira et al., 2021). Ferreira et al. recently showed that an interaction between dynein and endophilin is necessary for FEME; in short, chemical inhibition of dynein with cilibrevin-D prevented FEME in cultured neurons (Ferreira et al., 2021). Actin has also been shown to interact with endophilin during EPA formation (Renard et al., 2015; Tyckaert et al., 2022), and actin depolymerization at the PM is necessary for the completion of FEME (Tyckaert et al., 2022; Chakrabarti et al., 2021). In addition, the endocytic scission protein dynamin is required for FEME (Boucrot et al., 2015). For EPA scission from the PM, specific interactions between dynamin, endophilin, actin, dynein, and members of the SNARE complex are essential (Casamento and Boucrot, 2020; Boucrot et al., 2015; Wang et al., 2023). After EPA scission is completed, dynein carries the EPA on MTs to early endosomes for further processing (Casamento and Boucrot, 2020; Renard et al., 2015; Fu et al., 2011). Cyclin-dependent kinase-5 (CDK5) and glycogen synthase kinase-3 β (GSK3 β ) have been shown to negatively regulate FEME, as CDK5 inhibition by the drug dinaciclib or GSK3 β inhibition by the drug CHIR99021 significantly enhanced FEME activity in hippocampal neurons (Ferreira et al., 2021). The authors of the study hypothesized that phosphorylation of some component of EPAs by CDK5 or GSK3 β prevented FEME, and that dephosphorylation of EPAs was essential to the completion of FEME. FEME, therefore, involves the following sequence (I) a receptor-ligand interaction or some other stimulus signals internalization (Boucrot et al., 2015); (II) endophilin and the cargo to be internalized are recruited (Chan et al., 2018); (III) the FEME carrier is formed via an interaction between endophilin, actin, dynein, and synaptotagmin (Simunovic et al., 2017; Watanabe et al., 2018); (IV) dynamin-mediated EPA scission occurs (Renard et al., 2015; Imoto et al., 2022), and (V) EPA is trafficked via dynein on MTs to early endosomes for sorting (Ferreira et al., 2021; Simunovic et al., 2017). The step-by-step FEME-mediated internalization is depicted in Figure 2.

Endophilin plays an essential role in the regulated endocytosis of ion channels such as AMPAR, facilitating their role in the regulation of LTP and LTD (Chowdhury et al., 2006; Zhang et al., 2017; Stokes et al., 2025). The zeta inhibitory peptide, which impairs memory maintenance and LTP in rat hippocampal neurons (Pastalkova et al., 2006), was recently shown to cause AMPAR internalization in an EndoA2-dependent manner (Stokes et al., 2025). This effect was also observed through the application of competitive binding peptides (Stokes et al., 2025), which inhibit the interaction between EndoA2 and AMPAR (Stokes et al., 2025). Although dynamin does not play a role in the constitutive trafficking of AMPARs (Glebov et al., 2015), dynamin has been shown to interact with endocytic vesicles containing AMPARs (Fiuza et al., 2017). Similarly, dynein is essential for triggered AMPAR internalization, but not for constitutive AMPAR internalization (Kim and Lisman, 2001; Lin et al., 2000). Actin depolymerization is also necessary during AMPAR internalization, and actin stabilization by drugs like jasplakinolide prevents AMPAR internalization (Zhou et al., 2001). Although triggered AMPAR internalization and FEME both require dynamin-mediated scission, actin depolymerization, and interactions with dynein, AMPAR internalization has yet to be identified as FEME.

In addition to their integral role in exocytosis, SNARE complex proteins are also regulators of various types of endocytosis. In particular, substantial evidence exists for the involvement of Syt11 in endocytosis. Shimojo et al. recently showed that Syt11 plays a key role in the regulation of endocytosis and endosomal signaling in the basal forebrain and is essential for neurodevelopment and synaptic plasticity (Shimojo et al., 2019). In hippocampal neurons, Syt11 was demonstrated to regulate bulk endocytosis in a dynamin-dependent manner by preventing membrane invagination and vesicle formation (Wang et al., 2016). In cortical astrocytes, Syt11 was reported to stabilize caveolin to prevent caveolae-mediated endocytosis (Yan et al., 2020). In striatal neurons, Syt11 was found to inhibit the endocytic recycling of SVs to prevent excessive dopamine release (Wang et al., 2018). Recently, Syt11 was also shown to colocalize with EndoA1, and to inhibit SV endocytosis by inhibiting EndoA1 function in hippocampal neurons (Wang et al., 2023).

Endophilin and Syt11 are also implicated in the internalization of other ion channels involved in LTP and LTD. L-VGCC internalization requires the formation of a complex between the channel and endophilin (Chen et al., 2003; Lai et al., 2005); by blocking complex formation using an inhibitory peptide, L-VGCC internalization could be prevented, causing hippocampal neuron overstimulation (Chen et al., 2003; Lai et al., 2005). Recently, presynaptic N-VGCC internalization was also shown to be mediated through endophilin (Chen et al., 2003; Tong et al., 2017; Oh et al., 2023). In the neural synapses of *C. elegans*, the endophilin analogue UNC-57 (which stands for ‘uncoordinated’) is required for SV endocytosis (Schuske et al., 2003). UNC-57 was also shown to mediate the internalization of presynaptic N-VGCCs in *C. elegans* neurons (Tong et al., 2017; Oh et al., 2023). Moreover, viral knockdown of UNC-57 prevented N-VGCC internalization (Tong et al., 2017; Oh et al., 2023). Recently, Syt11 was shown to stabilize presynaptic N- and P/Q-VGCCs by preventing their constitutive and evoked internalization (Trovò et al., 2024). Induced knockout (KO) of Syt11 in hippocampal neurons led to an increase in presynaptic N- and P/Q-VGCC internalization and a reduction in neurotransmitter release (Trovò et al., 2024). CDK5 was recently shown to enhance and stabilize presynaptic N-VGCC activity via phosphorylation of collapsin response mediator protein 2 (CRMP2) (Brittain et al., 2012; Moutal et al., 2016; Gomez et al., 2020). CRMP2 is a microtubule-binding protein involved in cytoskeletal regulation; in addition to being essential for the regulation of neuronal polarity (Sumi et al., 2018), CRMP2 has been shown to play roles in the regulation of neuronal ion channel trafficking and the stabilization of synapses (Ip et al., 2014). Given that Syt11 interacts with endophilin to prevent FEME, and that Syt11 and CDK5 stabilize N-VGCC activity in presynaptic terminals, FEME may therefore play a role in the internalization of VGCCs.

VGSC internalization has also been implicated in the regulation of hippocampal activity (Fréal et al., 2023). The axon initial segment is the final synaptic integration site for AP generation (Kole and Stuart, 2012; Leterrier, 2018). VGSCs, especially Na_V_1.2 and Na_V_1.6, are clustered at the axon initial segment, and this high concentration of VGSCs is essential to the integration of excitatory potentials and the initiation of AP firing (Kole and Stuart, 2012; Leterrier, 2018). The balance between internalization and externalization of VGSCs at the axon initial segment is essential for homeostasis in pyramidal neurons of the hippocampus (Jamann et al., 2021). While an increase in presynaptic activity in these neurons caused a rapid shortening of the axon initial segment and internalization of VGSCs, a decrease in presynaptic activity caused a rapid lengthening of the segment and externalization of VGSCs (Jamann et al., 2021; Kuba et al., 2010). Hence, VGSC internalization in hippocampal neurons is dependent on presynaptic activity, and this process functions to maintain tightly regulated activity levels (Fréal et al., 2023; Jamann et al., 2021). This ion channel internalization process may also be mediated by Syt1 (Sampo et al., 2000). Sampo et al. showed that Syt1 interacts with Na_v_1.2, but not with syntaxin-1, SNAP-25, or VAMP-2 (Sampo et al., 2000). Moreover, VGSC internalization was not paired with VGCC internalization, and these processes did not utilize the same SNARE complex proteins in the same region (Sampo et al., 2000). VGSC internalization at the axon initial segment may therefore be regulated by a different endocytic pathway. FEME was recently shown to occur at the axon initial segment in hippocampal neurons, suggesting that it may regulate major endocytic pathways at this location (Fei et al., 2026). Though the channels that undergo FEME-mediated internalization at the axon initial segment have not been definitively identified, the rapidity with which VGSCs are internalized following an increase in presynaptic activity suggests that FEME could be responsible.

As noted above, ion channel internalization also helps regulate excitability in MNCs during recovery from hyperosmotic stimuli. In MNCs, recovery from osmotically-induced somatic hypertrophy and ΔN-TRPV1 channel internalization require dynamin-mediated endocytosis (Shah et al., 2014; Haan et al., 2023). These recovery processes occur within 30 min of a return to isosmotic solution at room temperature (Shah et al., 2014; Haan et al., 2023). Hypertrophy and ion channel translocation have been shown to depend on the activation of both phospholipase C (PLC) and protein kinase C (PKC) (Shah et al., 2014; Haan et al., 2023). PKC is activated by diacylglycerol (DAG), which is generated during the PLC-mediated conversion of phosphatidylinositol 4,5-bisphosphate (PIP_2_) into inositol triphosphate (IP_3_) and DAG (Ryu et al., 1990; Wu et al., 1999). Syt11, EndoA1, and CDK5 have all been shown to be expressed in MNCs (Yue et al., 2006; Thompson et al., 2023; Johnson et al., 2015). Because CDK5 is activated by PKC in cortical neurons (Zhao et al., 2009), we postulate that CDK5 is also activated by PKC in MNCs. The recovery from hypertrophy also involves a reorganization of the actin and MT cytoskeleton through depolymerization (Barad et al., 2020; Hicks et al., 2020). We postulate that CDK5 may negatively regulate ion channel internalization in MNCs, possibly by preventing Syt11 and EndoA1 from internalizing vesicles containing channels following a reorganization of the cytoskeleton. While this has been observed in FEME, it has not yet been studied in this context.

## Similar mechanisms govern translocation and internalization in peripheral nerves

Many of the mechanisms identified in central neurons are also employed in peripheral neurons, with proteins like TRPV1, endophilin, and SNARE proteins playing an important role. In nociceptive dorsal root ganglion (DRG) neurons, repetitive noxious stimuli (e.g., heat and capsaicin (Bevan et al., 2014; Gavva et al., 2004)) evoke TRPV1 translocation to the PM (Malek et al., 2015; Ma et al., 2017). These TRPV1 channels respond to the noxious stimuli, enhancing nociceptive transmission and pain sensitivity (i.e., enhance excitability) (Malek et al., 2015; Wang, 2008). Hence, translocation of TRPV1 makes the DRG neurons more susceptible to noxious stimuli, which further sensitizes them (Malek et al., 2015). In DRG neurons, translocation was demonstrated to depend on Ca^2+^ influx and SNARE exocytic membrane fusion (Camprubí-Robles et al., 2009); specifically, interactions with Syt1 and Syt9 (Toro et al., 2011; Meng et al., 2009), and with syntaxin-1 and SNAP-25 (Meng et al., 2016). As stated above, Syt1, syntaxin-1, and SNAP-25 are SNARE isoforms that are also involved in AMPAR and N-VGCC translocation. Although an increased sensitivity to noxious stimuli is thought to be a protective mechanism (Gao et al., 2024), prolonged sensitivity can lead to the development of hypersensitive pain states like neuropathic pain (Malek et al., 2015; Gao et al., 2024). In this context, the internalization of ion channels (e.g., TRPV1 from the somata of DRG neurons) is essential to maintaining appropriate excitability, and a dysfunction in this process has been implicated in pathophysiological states such as inflammatory pain (Malek et al., 2015; Liu et al., 2019). Na_V_1.7 has been identified as a key VGSC in DRG neurons, where it is responsible for the propagation of various pain-related signals (Dib-Hajj et al., 2007; Black et al., 2012; Hoffmann et al., 2018), and especially for mediating the response to inflammatory pain (Nassar et al., 2004; Emery et al., 2016; Kwon et al., 2021). Inflammation-mediated translocation of Na_V_1.7 to the PM has also been shown to occur rapidly, is associated with increased excitability and the propagation of pain signals (Higerd-Rusli et al., 2022; Tyagi et al., 2024), and may be mediated by Syt2 (Kanellopoulos et al., 2018).

Fialho et al. recently demonstrated that EndoA1 and synaptojanin-1—another protein involved in FEME (Watanabe et al., 2018)—are both necessary for SV recycling in nociceptive neurons (Pessano Fialho et al., 2025). In this study, they used siRNA to knock down the function of either EndoA1 or synaptojanin-1 (Pessano Fialho et al., 2025). They found that knockdown of EndoA1 or synaptojanin-1 prevented nociceptive signal transmission by preventing SV recycling (Pessano Fialho et al., 2025). In turn, inhibition of SV recycling reduced the activity of pro-nociceptive mechanisms, including thermally-induced TRPV1 activation (Pessano Fialho et al., 2025). Although this study did not demonstrate that TRPV1 channels were endocytosed in the SVs mediated by EndoA1, TRPV1 channels were previously localized to SVs (enabling them to be involved in secretory ion channel translocation), and are a regulator of SV recycling (Goswami et al., 2010). It is therefore possible that inhibition of recycling and of the response to thermal stimuli (as reported in the Fialho et al. study) were caused (in part) by internalization of SVs containing TRPV1. Interestingly, CDK5 prevents dynamin-mediated and clathrin-mediated TRPV1 endocytosis in DRG neurons by phosphorylating a clathrin adaptor protein called AP_2_ (Liu et al., 2019), which is a protein that is also involved in AMPAR endocytosis (Fiuza et al., 2017). FEME may also be the process regulating TRPV1 internalization in nociceptors. The observed similarities between AMPAR internalization, TRPV1 internalization, and FEME suggest that there may be a common underlying mechanism for triggered ion channel internalization.

## Conclusion and insights

In this review, we highlight commonalities present in the exocytosis and endocytosis of specific and well-described forms of neuroplastic and homeostatic ion channel translocation. LTP and LTD in learning and memory are the classic examples of neuroplastic translocation to and from the PM to modulate synaptic strength (Lynch, 2004; Pastalkova et al., 2006; Collingridge et al., 2010). However, neuroplastic changes also occur in other processes like motor coordination after muscle or nerve injury (Gokeler et al., 2019; Heuninckx et al., 2008; Byl et al., 2003; Izadi-Najafabadi et al., 2020). We also highlight homeostatic translocation in MNCs, although it is important to note that other examples exist, including that of regulatory TRP and Ca^2+^ channel translocation to modulate neurodevelopment (Turrigiano, 1999; Turrigiano and Nelson, 2004; Desai, 2003). A dysfunction of this process can lead to the development of diseases such as epilepsy (Bozzi et al., 2012; Tien and Kerschensteiner, 2018; Wolfart and Laker, 2015). The degree and type of ion channel translocation is also dependent on the lipid composition of the PM (Bastiaanse et al., 1997; Rosenhouse-Dantsker et al., 2012). Subcellular compartment-specific pathways for the recycling and degradation of internalized ion channels also play a role in evoked ion channel translocation (Donato et al., 2019; Terenzio et al., 2017). However, in-depth discussions of the effects of PM lipid composition and subcellular sorting on ion channel translocation are outside the scope of this review and thus were not expanded upon.

Our understanding of the importance of both endophilin and FEME to the regulation of neuronal activity through ion channel trafficking has grown substantially in the last 10 years. The growing body of data strongly suggests that endophilin and FEME play pivotal roles in maintaining normal neuronal function. For example, CDK5 has been shown to inhibit post-synaptic L-VGCCs (Loya-López et al., 2020), and to potentiate NMDAR signals and stabilize their presence on the post-synaptic PM (Li et al., 2001; Chergui et al., 2004; Fischer et al., 2003; Hernandez et al., 2016). As discussed earlier, L-VGCCs can inhibit AMPAR translocation to reduce LTP (Hiester et al., 2017), while NMDAR activity can enhance LTP (Lüscher and Malenka, 2012). Hence, CDK5 inhibition of L-VGCCs and CDK5 potentiation of NMDARs both enhance LTP. The emerging role of endophilin as a key mediator of ion channel internalization during the regulation of neuropathic sensitization provides evidence that its importance in translocation extends beyond the central nervous system (Pessano Fialho et al., 2025; Xie et al., 2024).

Syt11 dysfunction is associated with excessive dopamine release (Wang et al., 2018), and has been linked to Parkinson’s Disease (PD) (Wang et al., 2018; Sesar et al., 2016; Ng and Cao, 2024; Wang et al., 2020). EndoA1 dysfunction was also identified as a risk factor for PD development in genome-wide associated studies (Chang et al., 2017; Nguyen et al., 2019). Furthermore, a recent study has demonstrated that a dysfunctional interaction between EndoA1 and Syt11 contributes to synaptic neurodegeneration in PD (Gcwensa et al., 2021). Many patients with PD experience dysfunctional osmoregulation (Yang et al., 2020; Guo et al., 2022), and osmoregulatory issues have been identified as a potential contributor to the progression of PD (Yang et al., 2020; Guo et al., 2022; Morales et al., 2007). The emerging roles of FEME-related proteins (e.g., EndoA1 and Syt11) in the pathophysiology of neurodegenerative diseases like PD (Sesar et al., 2016; Ng and Cao, 2024; Yang et al., 2023), and Alzheimer’s Dementia (AD) (Yu et al., 2018; Ren et al., 2008), supports the importance of the regulation of ion channel translocation in neurodegenerative disease pathophysiology. The elucidation of the mechanisms of ion channel translocation may therefore reveal novel potential drug targets that may prove useful in the development of better pharmacotherapeutic agents. Despite many breakthroughs in PD treatment in the last 15 years, long-term treatment of PD remains notoriously difficult (Lee and Yankee, 2021; Bloem et al., 2021). The discovery of novel target proteins—such as EndoA1 and Syt11 (Ng and Cao, 2024)—that may be significant contributors to PD and AD pathophysiology (Ng and Cao, 2024; Yang et al., 2023; Yu et al., 2018), and possibly contributors to the pathologies of other neurodegenerative diseases, should help guide drug discovery and the development of better drugs.
